# Statin for Tuberculosis and Pneumonia in Patients with Asthma–Chronic Pulmonary Disease Overlap Syndrome: A Time-Dependent Population-Based Cohort Study

**DOI:** 10.3390/jcm7110381

**Published:** 2018-10-24

**Authors:** Jun-Jun Yeh, Cheng-Li Lin, Chung-Y. Hsu, Zonyin Shae, Chia-Hung Kao

**Affiliations:** 1 Department of Family and Chest Medicine, Ditmanson Medical Foundation Chia-Yi Christian Hospital, Chiayi 60002, Taiwan; 2 Department of Childhood Education and Nursery, Chia Nan University of Pharmacy and Science, Tainan 71710, Taiwan; 3 Department of Family Medicine, China Medical University, Taichung 40447, Taiwan; 4 Department of Nursing, Mei-Ho University, Pingtung 91252, Taiwan; 5 Management Office for Health Data, China Medical University Hospital, Taichung 40447, Taiwan; orangechengli@gmail.com; 6 College of Medicine, China Medical University, Taichung 40447, Taiwan; 7 Graduate Institute of Biomedical Sciences and School of Medicine, College of Medicine, China Medical University, Taichung 40447, Taiwan; hsuc@mail.cmuh.org.tw; 8 Department of Computer Science and Information Engineering, Asia University, Taichung 40447, Taiwan; zshae1@gmail.com; 9 Department of Nuclear Medicine and PET Center, China Medical University Hospital, Taichung 40447, Taiwan; 10 Department of Bioinformatics and Medical Engineering, Asia University, Taichung 40447, Taiwan

**Keywords:** tuberculosis, asthma–chronic pulmonary disease overlap syndrome, statins, pneumonia

## Abstract

We investigated the effects of statins on tuberculosis (TB) and pneumonia risks in asthma–chronic pulmonary disease overlap syndrome (ACOS) patients. We extracted data of patients diagnosed as having ACOS during 2000–2010 from the Taiwan National Health Insurance Research Database and divided them into statin users and nonusers. All study participants were followed up from the index date until death, withdrawal from insurance, or TB and pneumonia occurred (31 December 2011). The cumulative TB and pneumonia incidence was analyzed using Cox proportional regression analysis with time-dependent variables. After adjustments for multiple confounding factors including age, sex, comorbidities, and use of medications [statins, inhaled corticosteroids (ICSs), or oral steroids (OSs)], statin use was associated with significantly lower TB [adjusted hazard ratio (aHR) 0.49, 95% confidence interval (CI) 0.34–0.70] and pneumonia (aHR 0.52, 95% CI 0.41–0.65) risks. Moreover, aHRs (95% CIs) for statins combined with ICSs and OSs were respectively 0.60 (0.31–1.16) and 0.58 (0.40–0.85) for TB and 0.61 (0.39–0.95) and 0.57 (0.45–0.74) for pneumonia. Thus, statin users had lower TB and pneumonia risks than did nonusers, regardless of age, sex, comorbidities, and ICS or OS use. Pneumonia risk was lower among users of statins combined with ICSs or Oss and TB risk was lower among the users of statins combined with OSs.

## 1. Introduction

Pneumonia, a leading cause of mortality, places a heavy burden on both communities and healthcare systems [1,2]. Tuberculosis (TB) is a prevalent nosocomial infection, which often results in disability [3]. Chronic airway diseases, such as asthma and chronic obstructive pulmonary disease (COPD), are associated with pneumonia [4,5].

Hyperlipidemia was found to be a predisposing factor for pneumonia in one study [6] but was reported to be a factor potentially preventing TB in another study [7]. Cholesterol in the foamy macrophage plays a critical role in the reactivation of latent TB and the mentation of a persistent chronic TB infection. Phagosomal maturation and autophagy are responsible for the elimination of the bacteria and TB bacilli. A statin-mediated reduction in cholesterol levels within the phagosomal membranes potentially counteract the TB-induced inhibition of phagosomal maturation and promote host-induced autophagy, thereby augmenting protection against TB [8]. The role of cholesterol in pneumonia and TB development has been widely debated [9].

Statins exert anti-inflammatory effects on system inflammation and airway diseases such as asthma and COPD [10,11,12]. Pneumonia may increase an individual’s chances of contracting asthma and COPD later in life. Moreover, this condition is particularly responsible for the acute exacerbation of these two diseases. Fewer statin-using chronic respiratory disease patients [13] developed pneumonia than those not using statin [14]. Because bacteria attach to cell surfaces, cholesterol may be vital for the uptake and internal transport of certain bacteria [15]. Statins attenuate cholesterol levels, resulting in a lower chance of bacteria entering the cell to reproduce and destroy lung tissue. Moreover, cholesterol-lowering statins boost bacteria-killing cells [16]. By using human airway epithelial cells as an in vitro model, Statt et al. reported that prior exposure to physiological nanomolar serum concentrations of simvastatin (range 10–1000 nM) confers a significant cellular resistance to the cytotoxicity of pneumolysin, a pore-forming toxin that is the main virulence factor of *Streptococcus pneumonia* [17]. Therefore, hyperlipidemia with statin use may reduce the risk of pneumonia [18].

Asthma–chronic pulmonary disease overlap syndrome (ACOS) has components of both asthma and COPD. ACOS patients may have higher pneumonia [19] and TB [20] risks in the later course than do COPD patients [21]. Statins have anti-inflammatory and immunomodulatory properties, potentially resulting in reduced reactivation of latent TB; statins modulate T cell count and cytokine levels during pneumonia [22,23]. Few studies have reported on the relationship of statin, inhaled corticosteroid (ICS), or oral steroid (OS) use with TB or pneumonia in ACOS patients [24,25]. We explored this relationship in reference to the general population [26].

## 2. Methods

### 2.1. Data Source

This retrospective cohort study was conducted using data from the Longitudinal Health Insurance Database of one million enrollees extracted from the Taiwan National Health Insurance (NHI) Research Database (NHIRD) between 2000 and 2011 [27]. The NHI program, implemented since 1995, provides comprehensive medical care to nearly all of Taiwan’s population and covers the registry of beneficiaries, ambulatory care, inpatient care, prescriptions, and other medical services. This program has been described in numerous studies; for instance, by using 2005–2009 data from the NHIRD [28], Cheng et al. reported no association between cumulative statin use and intracranial hemorrhage risk in patients without a past history of stroke. This experience was incorporated into the 2017 Taiwan lipid guidelines for high risk patients [29]. Similarly, our study can provide baseline trends useful for further research on statins or other hyperlipidemia drug use [30]. International Classification of Diseases, Ninth Revision, Clinical Modification (ICD-9-CM) codes were used to define the diseases. This research was approved by the Research Ethics Committee at China Medical University and Hospital, Taiwan (27 June 2018, CMUH104-REC2-115-CR3).

### 2.2. Patients

The patients aged ≥18 years diagnosed as having ACOS [i.e., COPD (ICD-9-CM codes 491, 492, and 496) and/or asthma (ICD-9-CM code 493)] between 2000 and 2010 were selected. The date of ACOS diagnosis was defined as the index date [26,31]. Patients aged <18 years or having baseline diagnoses of TB (ICD-9-CM codes 010–018) or pneumonia (ICD-9-CM codes 481–486) were excluded. Patients who did and did not receive statin treatment were defined as statin users and nonusers, respectively.

### 2.3. ACOS, Pneumonia, and TB Patient Validation

In Taiwan, COPD is diagnosed through chest X-ray (CXR; 84.7%), computed tomography (CT; 39.4%), pulmonary function test (PFT; 58.44%), and smoking status (82.9%) [32]. Su et al. report high sensitivity of these asthma (92.0%) and COPD (86.2%) [33] diagnosis methods. ACOS patients undergo these diagnostic procedures often because medical services for diseases, such as pneumonia and TB, are easily available [21,23]. Moreover, in the 12 months following the index date, ACOS patients undergo CXR, CT, and PFT more often than do COPD or asthma patients—which supports our speculations [26]. Thus, extracting data of ACOS patients from among COPD and asthma patients in the NHIRD is reasonable.

We validated ACOS patients based on the following information: (1) Clinical manifestation in form of clinical symptoms or signs, such as chronic productive cough for 3 months in 2 successive years in a patient, wheezing, and dyspnea, aid early ACOS detection [34]; (2) Imaging, particularly CXR, has a 90% sensitivity and 98% specificity for emphysema [35] (51.0% of ACOS patients undergo CXR [26]); (3) ICS or OS use is a useful tool for confirming ACOS patients [36]. We used the anatomical therapeutic chemical code R03 for confirming of ACOS (24.0% and 76.0% ICS and OS use, respectively). Similarly, Su et al. reported ICS use in 53.48% of ACOS patients during follow-up [33], and Shantakumar et al. reported ICS and OS use in 46.1% and 85.5% of ACOS patients during a 1-year follow-up [26]. In the current study, 11,129 (98.9%) of 11,256 ACOS patients undertook COPD-, asthma-, TB-, or pneumonia-related diagnostic tests, such as CXR, PFT, CT, or immunoglobulin E levels, validating our ACOS cohort from the NHIRD.

In Taiwan, TB is confirmed through sputum culture and smear, CXR with clinical symptoms and signs, and therapeutic response to anti-TB drugs [37]. Su et al. found that among 433 patients diagnosed as having TB, 326 received at least two anti-TB drugs for 4 weeks and were selected for validation of the TB definition. Of these, 314 were confirmed as having TB [38] at a sensitivity of 96.3%. Pneumonia was confirmed through sputum smear and culture, blood culture, elevation in serum blood titer or urinary antigen levels, CXR, and therapeutic response to antibiotics [39]. Overall, 284 of 300 inpatients and 277 of 300 outpatients were validated as having pneumonia, with a sensitivity was 94.7% and 92.3%, respectively.

Therefore, the TB and pneumonia codes in the NHIRD are typical reflect the real-world diagnosis of TB and pneumonia in Taiwan.

### 2.4. Outcome Measurement and Potential Comorbidities

The endpoint of this study was TB and pneumonia incidence. All study participants were followed up from the index date until death, withdrawal from insurance, or the study endpoint (31 December 2011). We also included potential TB- or pneumonia-related comorbidities, including sleep disorders, diabetes, hypertension, hyperlipidemia, mental disorders, alcohol-related illnesses, and chronic kidney disease. Other medications such as ICSs and OSs were included if they were potentially correlated with TB or pneumonia development.

### 2.5. Propensity Score Matching

Statin users and nonusers were matched at a 1:1 ratio based on their propensity scores. We used logistic regression to calculate the propensity score for each patient by estimating assignment probability according to the baseline variables of age, sex, comorbidities (sleep disorders, diabetes, hypertension, hyperlipidemia, mental disorders, alcohol-related illness, and chronic kidney disease), and use of other medications (ICSs and OSs). This ensured that a similar number of patients were included in both the cohorts with an equal probability to statin use.

### 2.6. Statistical Analysis

Differences in demographic distributions, baseline comorbidities, and medications between statin users and nonusers were compared using the chi-square test for categorical variables and Student’s *t* test for mean ages. The Kaplan–Meier method was used to measure the cumulative TB or pneumonia incidence among statin users and nonusers. The cumulative curves of the two cohorts were tested using the log-rank test. However, ACOS patients may have taken their prescription irregularly during the study period; this may have led to an overestimation of the drug effects. To reduce this bias, we used Cox proportional hazard models with time-dependent exposure covariates [40] to estimate the hazard ratios (HRs) and their 95% confidence intervals (CIs) for TB and pneumonia in statin users relative to nonusers. Next, aHRs were measured after adjustments for age, sex, comorbidities, and use of medications and stratification by age, sex, and ICS and OS use. Moreover, we performed sensitivity analysis by developing Cox models based on propensity score–matched cohorts to refine our results. All statistical analyses were performed using SAS (version 9.4 for Windows; SAS Institute, Inc., Cary, NC, USA). A two-tailed *p* of <0.05 was considered statistically significant.

## 3. Results

Table 1 demonstrates that compared with nonusers, statin users were slightly younger (62.7 ± 11.7 vs. 64.4 ± 14.8 years), with a higher female: male ratio, higher prevalence of comorbidities, and higher likelihood of ICS or OS use.

**Figure 1:**
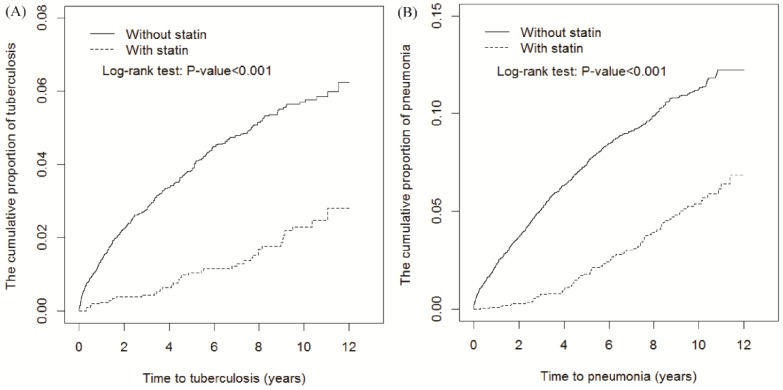
Cumulative TB (**A**) and pneumonia (**B**) incidence curves for statin users and nonusers.

The follow-up period was longer in statin users (8.18 ± 2.76 years) than in nonusers (5.99 ± 3.88 years; Table 2). By the end of the 12-year follow-up, cumulative TB and pneumonia incidence was 3.39% and 5.3% lower in statin users than in nonusers, respectively (both *p* < 0.001, log-rank test; Figure 1A,B respectively). Moreover, the time-dependent regression analysis after adjustment for age, sex, urbanization level, monthly income, occupation, comorbidities, and other medication use revealed that statin users had aHRs (95% CIs) of 0.49 (0.34–0.70) and 0.52 (0.41–0.65) for TB and pneumonia, respectively. TB and pneumonia incidence was respectively 2.21 and 5.23 per 1000 person-years in statin users and 6.96 and 13.7 per 1000 person-years in statin nonusers.

Table 3 lists TB and pneumonia incidence and HRs stratified by age and sex in both cohorts. Both male and female statin users had lower TB and pneumonia risks than did nonusers. Among patients aged ≥50 years, the TB and pneumonia risks were lower in statin users than in nonusers.

Table 4 presents TB and pneumonia incidence and HRs stratified by ICS and OS use in both cohorts. Compared with nonusers, statin use combined with ICS or OS use was significantly associated with lower pneumonia risk in statin users; however, statin use combined with OS use, but not ICS use, led to significantly lower TB risk.

The results of sensitivity analysis of propensity score–matched cohorts are listed in Table 5. TB incidence was 2.31 and 5.15 per 1000 person-years in statin users and nonusers, respectively. Statin users had a 0.37- and 0.47-fold lower TB and pneumonia risks, respectively (95% CI 0.25–0.56 and 0.36–0.61, respectively).

### 3.1. Sensitivity Analysis

Because most ACOS patients have higher readmission and mortality rates after discharge, we used a time-dependent model and long-term protective effects of statins to follow statin users (8.18 ± 2.76 years) and nonusers (5.99 ± 3.88 years). Long-term use was according to the higher cumulative dose recommended by the strict chronic prescription policy of Taiwan NHI. We further measured ICS and OS use among statin users to examine the effects of statins on TB and pneumonia risk further. Table 4 presents that the dichotomous effects of statins combined with ICSs or OSs noted in this study are similar to a forest plot.

### 3.2. Healthy User Bias

Considering “healthy user bias” is essential in the retrospective study of the pneumonia and TB outcomes of statin users. This bias is described as a higher awareness of health and a healthier lifestyle among statin users than that among nonusers. Therefore, statin users are potentially more likely to seek preventive health services, such as CXR, PFT, sputum culture, and vaccinations, particularly in the current ACOS cohort. However, measuring lifestyle factors, disease prevention behaviors, and drugs compliance in observational studies is difficult because lifestyle, income, and urbanization are confounded by alcohol-related diseases and sleep and mental disorders. To reduce the impact of the confounding healthy user bias, we measured alcohol-related diseases and sleep and mental disorders through individual insurance as a proxy to adjust for socioeconomic status. Propensity score matching, which included these as baseline variables, further reduced the healthy user bias. Furthermore, the factors associated with latent TB, such as nutrition (e.g., diabetes or hyperlipidemia), immune status, and steroid use, were included in the analysis. These statistical methods enable observational studies to simulate the results of randomized control trials.

### 3.3. Indication Bias of Statin Use with ICSs or OSs

The indication bias of statin may also be challenged in this study. In Taiwan, statins and ICSs are not available over the counter; the physicians’ decision to provide statin or ICS treatment should not only follow the treatment guidelines for the specific diseases but also the payment regulations by the NHI. If the prescriptions contravene Taiwan lipid guidelines and the Global Initiative for Chronic Obstructive Lung Disease (GOLD) guidelines, the NHI Administration may not only refuse to pay the medical fee but also punish the physicians with a maximum 100-fold penalty. This is because NHI is a single-payer compulsory insurance coverage policy and the NHI Administration has full authority to control all medical facilities and the concerned health care.

## 4. Discussion

The most crucial finding of this study was that statin-using ACOS patients had lower TB or pneumonia risk and that concurrent use of statin and ICSs or OSs was also associated a lower TB or pneumonia risk.

In asthma patients, statins can improve outcomes in combination with either ICSs or OSs [24,25]. Emergency rooms and hospitals are the sources of nosocomial infection; therefore, the cohort with superior outcomes and more infrequent visits may exhibit a lower incidence rate for nosocomial pneumonia and TB [19]. Moreover, the antibiotic effects of statins on TB during statin therapy are associated with a decreased active TB risk [41]—an observation supported by the effects of the duration of statin therapy in protecting against TB. A Taiwanese study using the NHIRD also reported that statin use is associated with a lower-than-usual TB risk [38].

According to an US study, ACOS patients can be classified as A (low risk, fewer symptoms, modified Medical Research Council (mMRC) score of 0–1, or COPD assessment test (CAT) score of <10), B (low risk, more symptoms, mMRC score of >2, or CAT score of >10), C (high risk, fewer symptoms, mMRC score of 0–1, or CAT score of <10), and D (high risk, more symptoms, mMRC score of >2, or CAT score of >10). The GOLD classifications characterizes A and B groups by a forced expiratory volume-1 second (FEV1) or forced vital capacity (FVC) of >50% with exacerbation of <1 per year and C and D groups by a FEV1 or FVC of <50% with exacerbation of >1 per year. Most ACOS patients are categorized in the B and D groups [34,42]. Poor lung function is associated with the development of TB or pneumonia classified in the D group (exacerbation of >1 per year) [31]. In a study, in COPD patients, statin use may have improved lung function, whereas it may have delayed decline in lung function in asthma patients [43]. These results are similar to those obtained by other studies, where after statin use in ACOS patients, some patients were reclassified from group D into group A (exacerbation of <1 per year), corresponding to the lowering of TB or pneumonia incidence [38,42]. These results are in accordance with those found in our study.

Improved lung function in COPD and asthma patients may be due to the anti-inflammatory effects of statin use [44,45,46]; moreover, concurrent use of ICSs or OSs with statin can lead to greater lung function improvement in an ACOS cohort [33]. These findings indicate the additive effects of statins and ICSs or OSs [47,48]; the combined use of statins and ICSs or OSs in ACOS patients may have therefore reduce the number of emergency hospital visits and exacerbation events [24] and thus the lower TB and pneumonia incidence, particularly in patients aged >50 years.

In another study, high long-term ICS or OS dosage was associated with an elevated TB risk in patients with asthma in the COPD cohort, with ICS use leading to the highest risk. Furthermore, exposure to ICSs was not associated with TB risk in the presence of OSs but with an increased TB risk in OS nonusers. The risk of TB among OS users has been well documented; moreover, the negative effects of ICSs have bed reported to be negligible, even at high doses. However, the previous studies have neither involved an ACOS cohort nor adjusted for statin and time dependency in their analyses. A population-based nested case–control study revealed that ICSs were not associated with TB development [49]; however, the benefits of ICSs and OSs may outweigh the risk of pneumonia in statin-using asthma patients [17,18]. ICSs and OSs demonstrated dichotomous effects on the incidence of TB and pneumonia in the ACOS cohort; hence, the effects of concurrent statin and ICS or OS treatment on TB development among patients in the ACOS cohort warrant further research.

The present work demonstrated that statins have beneficial anti-inflammatory and antioxidant effects beyond the reduction of cholesterol levels in TB or pneumonia patients. The accumulation of lipids in macrophages harboring TB is associated with persistent and phenotypic resistance to anti-TB drugs, leading to prolongation of treatment duration [8]. Statins play a role in the attenuation of both TB growth and infection-induced lipid accumulation in macrophages as well as the promotion of phagosomal maturation and autophagy. In line with the other studies, here, statins helped reduce TB development rate. Our study revealed that even with ICS or OS use, statin-using ACOS patients exhibited lower TB and pneumonia incidence. Determination of the correct statin administration dosage, timing, and duration among cohorts requires further confirmation.

By contrast, the reports mention of the statins without relationship or increase of the risk of pneumonia were found [50]. In a study, among 124,695 acute myocardial infarction (AMI) patients, 76,994 (61.9%) has statin prescription; of them, only 19,078 (15.3%) were diagnosed with pneumonia. Using an ordinary least squares method, the statin coefficient indicated that statin prescription was associated with a reduction in pneumonia incidence. However, the instrumental variables indicated that statin use was not associated with the reduction in pneumonia incidence. The study concluded that in AMI patients, the protective effect of statins against pneumonia is most likely the result of nonrandom treatment assignment (i.e., healthy user bias) [51].

In their meta-analysis, Chopra et al. revealed that two prospective studies (Majumdar and Yende et al.) found that statins did not reduce pneumonia-related mortality, but another prospective study (Chalmers et al.) found that they did [52]. Thus, Chopra et al. concluded that although statin use is associated with decreased pneumonia-related mortality, this effect weakens in important subgroups. According to Chalmers et al. statin use in animals reduced endothelial dysfunction and had antithrombotic effects, which improved pneumonia outcomes. Moreover, numerous clinical studies have suggested that statins may have a role in pneumonia prevention or prognosis improvement in hospitalized patients [53]. Only a randomized controlled study can completely explore the link among statins, pneumonia, and pneumonia-related mortality.

Our sensitivity analysis of propensity score–matched cohorts [54] revealed that statins were associated with a lower pneumonia and TB risks. This result may be explained by the different cohort and statistical methods used in the current research. We recommend using the optimal time, dose and duration of the statins may a conjunctive regimen [55] of the antibiotic, anti-TB or OSs/ICs in the ACOS cohort for attenuate the pneumonia sequelae or mortality [56]. These speculations warrant further large-scale research for confirmation.

### 4.1. Strengths

In traditional Kaplan–Meier or Cox regression analyses, a typical risk factor measured at baseline correlates with mortality thereafter. During follow-up, however, this may change: either the effect of a fixed baseline risk factor may vary over time, resulting in a weakening or strengthening of associations, or the risk factor itself may vary over time. A strength of the Cox model is its ability to encompass covariates this continually change. The reason for using time-dependent covariates is based on the underlying mechanisms of the Cox model; at the time of each event, the program compares current covariate values of the event subject to the current values of all others at risk at that given time. Time-dependent variables are those that can change in value over the course of the observation period [57]. Variables, such as medicine and the time at which patients receive ICSs or OSs and statins, may vary over time. Although researchers can hold the values of such variables fixed at a certain point in time (e.g., baseline), the changing values may have yielded different or even a more accurate analysis of data simply because we used as much data as possible. In this study, ACOS patients were identified based on COPD and medications used (e.g., ICSs) [26,36]. Moreover, pneumonia, TB, and ACOS diagnoses and follow-up were strongly validated by using other NHIRD-related study [26,31,37,58]. Furthermore, prescription and education policies regarding the use of ICSs or OSs and statins are well established in Taiwan [59,60]. Therefore, this NHIRD study (ACOS cohort and ICS, OS, or statin use) was representative of the real-world situation in Taiwan. We used propensity score matching to validate the sensitivity analysis and avoid selection bias.

### 4.2. Limitations

The following major points should be considered: (1) The modeling of a time-dependent covariate involves the selection of a function over time; the form this takes may not be obvious and requires considerable biological insight; (2) The selection of a complex functional form increases the possibility of excessive modeling and overfitting of a data set; (3) Numerous time-dependent covariates closely associated with the units under study are usually generated by that unit (e.g., ICS or OS and statin use) and alter the typical relationship between the hazard and survival functions; (4) time-dependent covariate models, except those under certain circumstances, do not allow the creation of individual predictive time-to-event curves; this is different from the Cox model, which only uses fixed covariate values; (5) Extreme caution must be exercised when modeling time-dependent exposure or treatments, particularly when changes in those variables are related to a participant’s health status, such as residential area (urban or rural) and insurance status (insured or uninsured). The opportunities inherent in time-dependent modeling (including the explicit relationship between longitudinal values and event occurrence) must be regarded considering potential biases, strong assumptions necessitated by the lack of other possible explanations, and need to select more complex functional forms for modeling [40]. Biochemical data of statin users and nonusers were unavailable in the NHIRD.

## 5. Conclusions

In ACOS patients, statin use was associated with lowest TB and pneumonia risks, regardless of age, sex, comorbidities, and ICS or OS use. Pneumonia risk was lower among users of statins combined with ICSs or Oss and TB risk was lower among the users of statins combined with OSs.

## Figures and Tables

**Table 1:** Demographic characteristics and clinical comorbidities.

	ACOS						*p*-Value
	Statin						
	All (*n* = 11256)		No (*n* = 9206)		Yes (*n* = 2050)		
	*n*	%	*n*	%	*n*	%	
Age, years							<0.001
<50	1960	17.4	1656	18.0	304	14.8	
50–64	3283	29.2	2502	27.2	781	38.1	
65+	6013	53.4	5048	54.8	965	47.1	
Mean ± SD ^a^	64.1	14.3	64.4	14.8	62.7	11.7	<0.001
Gender							<0.001
Women	5029	44.7	3969	43.1	1060	51.7	
Men	6227	55.3	5237	56.9	990	48.3	
Urbanization level ^§^							0.32
1 (highest)	2888	25.7	2367	25.7	521	25.4	
2	3116	27.7	2522	27.4	594	29.0	
3	1822	16.2	1513	16.4	309	15.1	
4 (lowest)	3430	30.5	2804	30.5	626	30.5	
Monthly income (NTD)							0.001
<15,840	3427	30.5	2872	31.2	555	27.1	
15,840–25,200	5379	47.8	4357	47.3	1022	49.9	
≥25,200	2450	21.8	1977	21.5	473	23.1	
Occupation							0.005
White collar	3996	35.5	3287	35.7	709	34.6	
Blue collar	4924	43.8	3967	43.1	957	46.7	
Others ^‡^	2336	20.8	1952	21.2	384	18.7	
Comorbidity							
Sleep disorder	5262	46.8	4127	44.8	1135	55.4	<0.001
Diabetes	2449	21.8	1740	18.9	709	34.6	<0.001
Hypertension	7958	70.7	6219	67.6	1739	84.8	<0.001
Hyperlipidemia	4662	41.4	3026	32.9	1636	79.8	<0.001
Mental disorders	6858	60.9	5443	59.1	1415	69.0	<0.001
Alcohol-related illness	1049	9.32	839	9.11	210	10.2	0.02
Chronic kidney disease	915	8.13	718	7.80	197	9.61	<0.001
Tobacco use	526	4.67	426	4.63	100	4.88	0.63
Coronary artery disease	5492	48.8	4232	46.0	1260	61.5	<0.001
Stroke	1986	17.6	1591	17.3	395	19.3	0.03
Acute respiratory failure	1405	12.5	1220	13.3	185	9.02	<0.001
Medication							
Inhaled corticosteroids (ICSs)	2706	24.0	2148	23.3	558	27.2	<0.001
Oral steroids (OSs)	8550	76.0	6876	74.7	1674	81.7	<0.001

Chi-square test; ^a^ Student’s *t* test; Monthly income in New Taiwan Dollar (NT$; NT$1 = US$0.03). ^§^ Urbanization level was categorized into four levels according to the population density of the residential area, with Level 1 and 4 being the most and least urbanized, respectively. ^‡^ Other occupational categories included primarily retired, unemployed, and low-income groups. ACOS: asthma–chronic pulmonary disease overlap syndrome.

**Table 2:** Cumulative tuberculosis and pneumonia incidence and hazard ratios of statin users and nonusers.

	Statin	
Variables	No (*n* = 9206)	Yes (*n* = 2050)
Tuberculosis		
Person-years	55,164	16,771
Follow-up time (year), Mean ± SD	5.99 ± 3.88	8.18 ± 2.76
Event, *n*	384	37
Rate	6.96	2.21
cHR (95% CI)	1 (Reference)	0.34 (0.25, 0.48) ***
aHR (95% CI) ^a^	1 (Reference)	0.49 (0.34, 0.70) ***
Pneumonia		
Person-years	54,267	16,649
Follow-up time (year), Mean ± SD	5.89 ± 3.41	8.12 ± 2.76
Event, *n*	742	87
Rate	13.7	5.23
cHR (95% CI)	1 (Reference)	0.40 (0.32, 0.50) ***
aHR (95% CI) ^a^	1 (Reference)	0.52 (0.41, 0.65) ***

Incidence in per 1000 person-years. ^a^ Data adjusted for age, sex, urbanization level, monthly income, occupation, comorbidities (sleep disorders, diabetes, hypertension, hyperlipidemia, mental disorders, alcohol-related illness, chronic kidney disease, tobacco use, CAD, stroke, and acute respiratory failure), and use of medication (ICSs and OSs). Abbreviations: cHR, crude hazard ratio; aHR, adjusted hazard ratio; ICSs, inhaled corticosteroids; OSs, oral steroids. *** *p* < 0.001.

**Table 3:** Age- and sex-stratified tuberculosis and pneumonia incidence and hazard ratios.

	**Men**		**Women**	
	**Statin**		**Statin**	
	**No (*n* = 5237)**	**Yes (*n* = 990)**	**No (*n* = 3969)**	**Yes (*n* = 1060)**
Tuberculosis				
No. of event	282	26	102	11
Incidence rate	9.30	3.29	4.11	1.24
cHR (95% CI)	1 (Reference)	0.38 (0.26, 0.57) ***	1 (Reference)	0.33 (0.18, 0.61) ***
aHR (95% CI) ^a^	1 (Reference)	0.53 (0.34, 0.81) **	1 (Reference)	0.41 (0.21, 0.79) **
Pneumonia				
No. of event	525	57	217	30
Incidence rate	17.6	7.27	8.87	3.41
cHR (95% CI)	1 (Reference)	0.43 (0.33, 0.57) ***	1 (Reference)	0.41 (0.28, 0.60) ***
aHR (95% CI) ^a^	1 (Reference)	0.54 (0.41, 0.73) ***	1 (Reference)	0.47 (0.32, 0.70) ***
	**Age < 50**		**Age ≥ 50**	
	**Statin**		**Statin**	
	**No (*n* = 1656)**	**Yes (*n* = 304)**	**No (*n* = 7550)**	**Yes (*n* = 1746)**
Tuberculosis				
No. of event	22	4	362	33
Incidence rate	1.84	1.50	8.38	2.34
cHR (95% CI)	1 (Reference)	0.85 (0.29, 2.48)	1 (Reference)	0.31 (0.21, 0.44) ***
aHR (95% CI) ^a^	1 (Reference)	0.74 (0.22, 2.49)	1 (Reference)	0.45 (0.31, 0.65) ***
Pneumonia				
No. of event	33	2	709	85
Incidence rate	2.78	0.75	16.7	6.08
cHR (95% CI)	1 (Reference)	0.29 (0.07, 1.21)	1 (Reference)	0.38 (0.30, 0.48) ***
aHR (95% CI) ^a^	1 (Reference)	0.20 (0.05, 0.91) *	1 (Reference)	0.52 (0.41, 0.67) ***

Incidence in per 1000 person-years. ^a^ Data adjusted for age, sex, urbanization level, monthly income, occupation, comorbidities (sleep disorders, diabetes, hypertension, hyperlipidemia, mental disorders, alcohol-related illness, chronic kidney disease, tobacco use, CAD, stroke, and acute respiratory failure), and use of medication (ICSs and OSs). Abbreviations: cHR, crude hazard ratio; aHR, adjusted hazard ratio; ICSs, inhaled corticosteroids; OSs, oral steroids. * *p* < 0.05, ** *p* < 0.01, *** *p* < 0.001.

**Table 4:** ICS and OS use-stratified tuberculosis and pneumonia incidence and hazard ratios.

	**With ICSs**		**Without ICSs**	
	**Statin**		**Statin**	
	**No (*n* = 2148)**	**Yes (*n* = 558)**	**No (*n* = 7058)**	**Yes (*n* = 1492)**
Tuberculosis				
No. of event	76	12	308	25
Incidence rate	5.33	2.54	7.53	2.07
cHR (95% CI)	1 (Reference)	0.49 (0.26, 0.89) *	1 (Reference)	0.31 (0.20, 0.46) ***
aHR (95% CI) ^a^	1 (Reference)	0.60 (0.31, 1.16)	1 (Reference)	0.44 (0.29, 0.67) ***
Pneumonia				
No. of event	151	26	591	61
Incidence rate	10.8	5.56	14.7	5.09
cHR (95% CI)	1 (Reference)	0.52 (0.34, 0.78) **	1 (Reference)	0.37 (0.29, 0.48) ***
aHR (95% CI) ^a^	1 (Reference)	0.61 (0.39, 0.95) *	1 (Reference)	0.48 (0.36, 0.63) ***
	**With OSs**		**Without Oss**	
	**Statin**		**Statin**	
	**No (*n* = 6876)**	**Yes (*n* = 1674)**	**No (*n* = 2330)**	**Yes (*n* = 376)**
Tuberculosis				
No. of event	237	34	147	3
Incidence rate	5.61	2.46	11.4	1.02
cHR (95% CI)	1 (Reference)	0.46 (0.32, 0.66) ***	1 (Reference)	0.10 (0.03, 0.32) ***
aHR (95% CI) ^a^	1 (Reference)	0.58 (0.40, 0.85) **	1 (Reference)	0.16 (0.05, 0.53) **
Pneumonia				
No. of event	534	79	208	8
Incidence rate	12.9	5.76	16.1	2.72
cHR (95% CI)	1 (Reference)	0.46 (0.36, 0.58) ***	1 (Reference)	0.19 (0.09, 0.38) ***
aHR (95% CI) ^a^	1 (Reference)	0.57 (0.45, 0.74) ***	1 (Reference)	0.26 (0.12, 0.53) ***

Incidence in per 1000 person-years. ^a^ Data adjusted for age, sex, urbanization level, monthly income, occupation, comorbidities (sleep disorders, diabetes, hypertension, hyperlipidemia, mental disorders, alcohol-related illness, chronic kidney disease, tobacco use, CAD, stroke, and acute respiratory failure), and use of medication (ICSs and OSs). Abbreviations: cHR, crude hazard ratio; aHR, adjusted hazard ratio; ICSs, inhaled corticosteroids; OSs, oral steroids. * *p* < 0.05, ** *p* < 0.01, *** *p* < 0.001.

**Table 5:** Overall tuberculosis and pneumonia incidence and hazard ratios in statin users vs. propensity score–matched statin nonusers.

	Statin	
Variables	No (*n* = 1984)	Yes (*n* = 1984)
Tuberculosis		
Person-years	14,570	16,040
Follow-up time (year), Mean ± SD	7.34 ± 3.25	8.08 ± 2.75
Event, *n*	75	37
Rate	5.15	2.31
cHR (95% CI)	1 (Reference)	0.38 (0.26, 0.57) ***
aHR (95% CI) ^a^	1 (Reference)	0.37 (0.25, 0.56) ***
Pneumonia		
Person-years	14,308	15,924
Follow-up time (year), Mean ± SD	7.21 ± 3.32	8.03 ± 2.75
Event, *n*	150	86
Rate	10.5	5.40
cHR (95% CI)	1 (Reference)	0.49 (0.38, 0.64) ***
aHR (95% CI) ^a^	1 (Reference)	0.47 (0.36, 0.61) ***

Incidence in per 1000 person-years. ^a^ Data adjusted for age, sex, urbanization level, monthly income, occupation, comorbidities (sleep disorders, diabetes, hypertension, hyperlipidemia, mental disorders, alcohol-related illness, chronic kidney disease, tobacco use, CAD, stroke, and acute respiratory failure), and use of medication (ICSs and OSs). Abbreviations: cHR, crude hazard ratio; aHR, adjusted hazard ratio; ICSs, inhaled corticosteroids; OSs, oral steroids. *** *p* < 0.001.
